# Adipocyte Fatty Acid-Binding Protein Promotes Palmitate-Induced Mitochondrial Dysfunction and Apoptosis in Macrophages

**DOI:** 10.3389/fimmu.2018.00081

**Published:** 2018-01-30

**Authors:** Hui Li, Yang Xiao, Lin Tang, Feng Zhong, Gan Huang, Jun-Mei Xu, Ai-Min Xu, Ru-Ping Dai, Zhi-Guang Zhou

**Affiliations:** ^1^Department of Anesthesiology, The Second Xiangya Hospital, Central South University, Changsha, China; ^2^Department of Metabolism and Endocrinology, The Second Xiangya Hospital, Central South University, Changsha, China; ^3^Department of Pharmacology and Pharmacy, Li Ka Shing Faculty of Medicine, The University of Hong Kong, Pokfulam, Hong Kong

**Keywords:** adipocyte fatty acid-binding protein, mitochondrial dysfunction, apoptosis, macrophage, palmitate

## Abstract

A high level of circulating free fatty acids (FFAs) is known to be an important trigger for macrophage apoptosis during the development of atherosclerosis. However, the underlying mechanism by which FFAs result in macrophage apoptosis is not well understood. In cultured human macrophage Thp-1 cells, we showed that palmitate (PA), the most abundant FFA in circulation, induced excessive reactive oxidative substance production, increased malondialdehyde concentration, and decreased adenosine triphosphate levels. Furthermore, PA treatment also led to mitochondrial dysfunction, including the decrease of mitochondrial number, the impairment of respiratory complex IV and succinate dehydrogenase activity, and the reduction of mitochondrial membrane potential. Mitochondrial apoptosis was also detected after PA treatment, indicated by a decrease in cytochrome *c* release, downregulation of Bcl-2, upregulation of Bax, and increased caspase-3 activity. PA treatment upregulated the expression of adipocyte fatty acid-binding protein (A-FABP), a critical regulator of fatty acid trafficking and lipid metabolism. Inhibition of A-FABP with BMS309403, a small-molecule A-FABP inhibitor, almost reversed all of these indexes. Thus, this study suggested that PA-mediated macrophage apoptosis through A-FABP upregulation, which subsequently resulted in mitochondrial dysfunction and reactive oxidative stress. Inhibition of A-FABP may be a potential therapeutic target for macrophage apoptosis and to delay the progress of atherosclerosis.

## Introduction

Macrophage apoptosis is an important characteristic of atherosclerosis development (1, 2). Numerous studies have revealed that macrophage apoptosis has distinct roles in the progression of atherosclerosis from early atherosclerotic lesions to advanced lesions. When efferocytosis, a process of removing dead cells, is compromised, macrophage apoptosis increases the size of the necrotic core and becomes a critical factor for plaque progression. Macrophage apoptosis can be induced by long-term exposure to high levels of free fatty acids (FFAs), a well-known risk factor predictive of cardiovascular death (3–5). Among the circulating FFAs, palmitate (PA) is the most abundant saturated fatty acid that promotes macrophage apoptosis and the conversion of stable plaques into vulnerable ones (6).

Although the exact mechanisms remain unclear, accumulating evidence suggests that PA can trigger oxidative stress and mitochondrial dysfunction, and eventually lead to apoptosis in various cell culture models (7–11). This effect may be partly due to the uncoupling of mitochondrial respiration induced by PA (11). PA administration results in apoptosis in cardiac myocytes, and it is accompanied by a decrease in mitochondrial membrane potential (Δψm), mitochondrial swelling, and an inhibition of carnitine palmitoyltransferase I activity (12). Studies in skeletal muscle cells, pancreatic β cells, and HepG2 cells also show that long-term exposure to FFAs has deleterious effects on mitochondria in multiple pathways, including increased reactive oxidative species (ROS) generation, impairment of mitochondrial dynamics, and biogenesis (7, 9, 13). However, knowledge of whether oxidative stress and mitochondrial damage mediates PA-induced macrophage apoptosis, and how they regulate it, is lacking.

Adipocyte fatty acid-binding protein (A-FABP; also known as aP2 or FABP4), is a novel adipokine that has recently received considerable attention because it integrates obesity, diabetes mellitus, and atherosclerosis (14, 15). The main function of A-FABP is to facilitate the intracellular trafficking of fatty acids between cellular compartments, thereby regulating cellular lipid metabolism and lipid signals (16). Despite its abundant expression in both adipocytes and macrophages, the proatherosclerotic effect of A-FABP is exclusively associated with its action on macrophages (17). The circulating A-FABP level correlates with unstable plaque characteristics and symptomatic lesions, and it is predictive for the occurrence of adverse cardiovascular events (18). The underlying mechanism of this link is further confirmed by recent findings that A-FABP also serves as a modulator of cell proliferation and apoptosis in macrophages and DU145 prostate cancer cells (19, 20). A deficiency of A-FABP suppresses caspase-3 activity and Bax and mitogen-activated protein kinase expression, all of which may be involved in the apoptosis process in different settings and cell types. The actions of A-FABP on ingestion, transportation, esterification, and β-oxidation of fatty acids have elicited considerable interest in further exploring the role of A-FABP in mitochondrial apoptosis. A-FABP overexpression reversed enhanced mitochondrial fatty acid oxidation and reduced the expression of mitochondrial biogenesis-related proteins, including peroxisome proliferator-activated receptor γ (PPARγ) coactivator 1-α and uncoupling protein 2, after induction by leptin in adipocytes (21). In addition, increasing evidence suggests that some of the FABP family members are endowed with the ability to regulate oxidative stress and mitochondrial fatty acid oxidation (22–26), which are closely related to mitochondrial function. However, it still unknown whether A-FABP is involved in the effect of PA on macrophage apoptosis and the related mitochondrial dysfunctions.

The aim of this study was to use a well-characterized *in vitro* model of human macrophages, Thp-1 cells, to determine whether FFAs cause mitochondrial dysfunction and consequently activate a mitochondrial apoptotic pathway in macrophages, and to determine whether A-FABP serves as a mediator in this signaling cascade.

## Results

### PA Induced the Upregulation of A-FABP Expression in Thp-1 Cells

**Figure 1:**
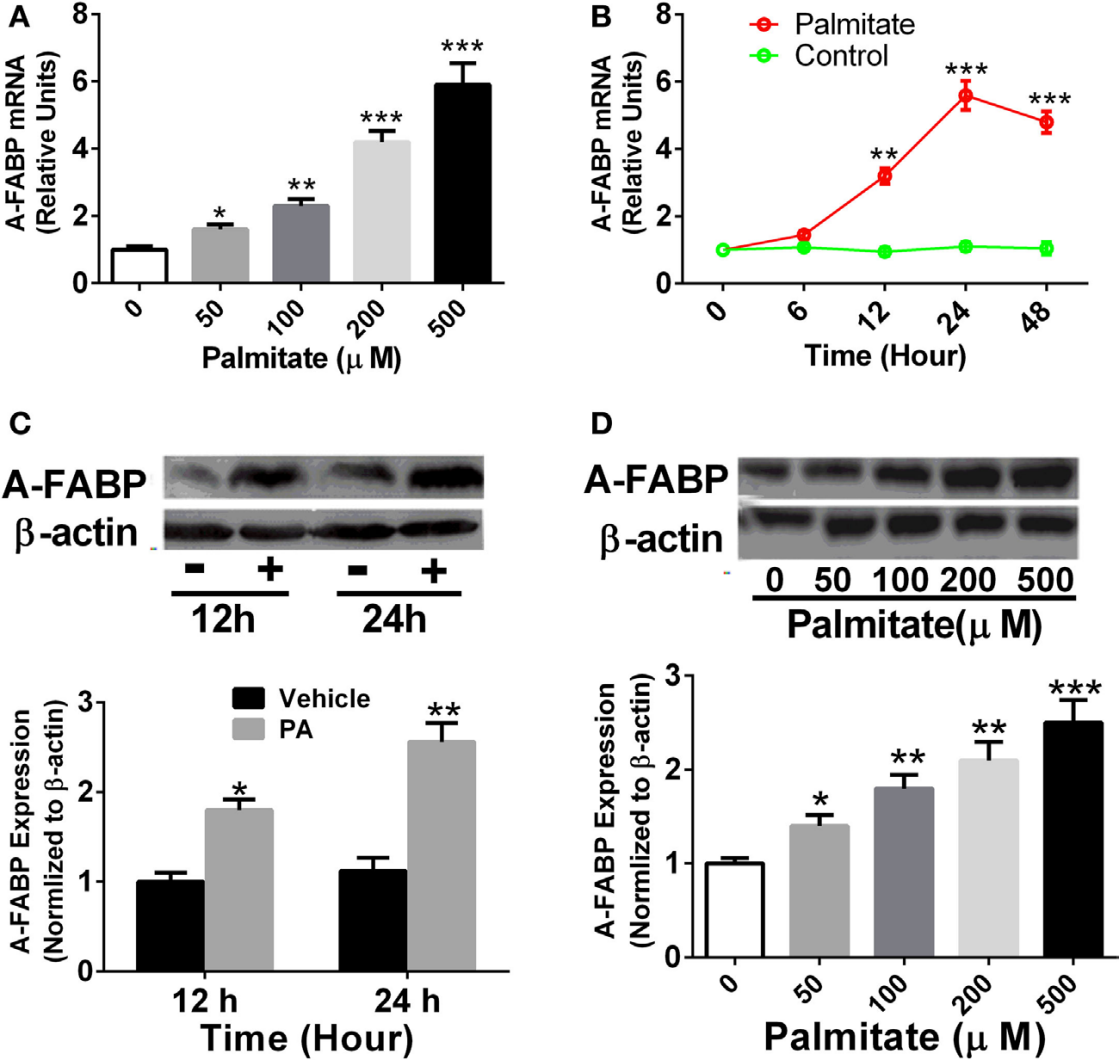
Palmitate (PA) potently induces adipocyte fatty acid-binding protein (A-FABP) expression in Thp-1 cells. **(A)** Thp-1 cells were stimulated with various concentrations of PA, and the mRNA level of A-FABP, examined 24 h after the treatment. **(B)** Time course of gene expression of A-FABP after 500 µM PA treatment. **(C)** Western blot of A-FABP (top panel) and its semiquantitative analysis (low panel) at 12 and 24 h post-treatment with 500 µM PA. **(D)** Western blot of A-FABP and its semiquantitative analysis at 24 h after various concentrations of PA treatment. *P < 0.05, **P < 0.01, ***P < 0.001 versus vehicle control (*n* = 3–5 for each group).

Thp-1 cells were treated with different concentrations of PA as indicated, and A-FABP mRNA and protein expression were examined. As shown in Figure 1, PA significantly increased the levels of A-FABP mRNA in a time- and dose-dependent manner (Figures 1A,B). Similarly, the protein expression of A-FABP was markedly increased in a time- and dose-dependent manner in the Thp-1 cells treated with PA (Figures 1C,D).

### Effects of A-FABP Inhibition on Cell Apoptosis Induced by PA

**Figure 2:**
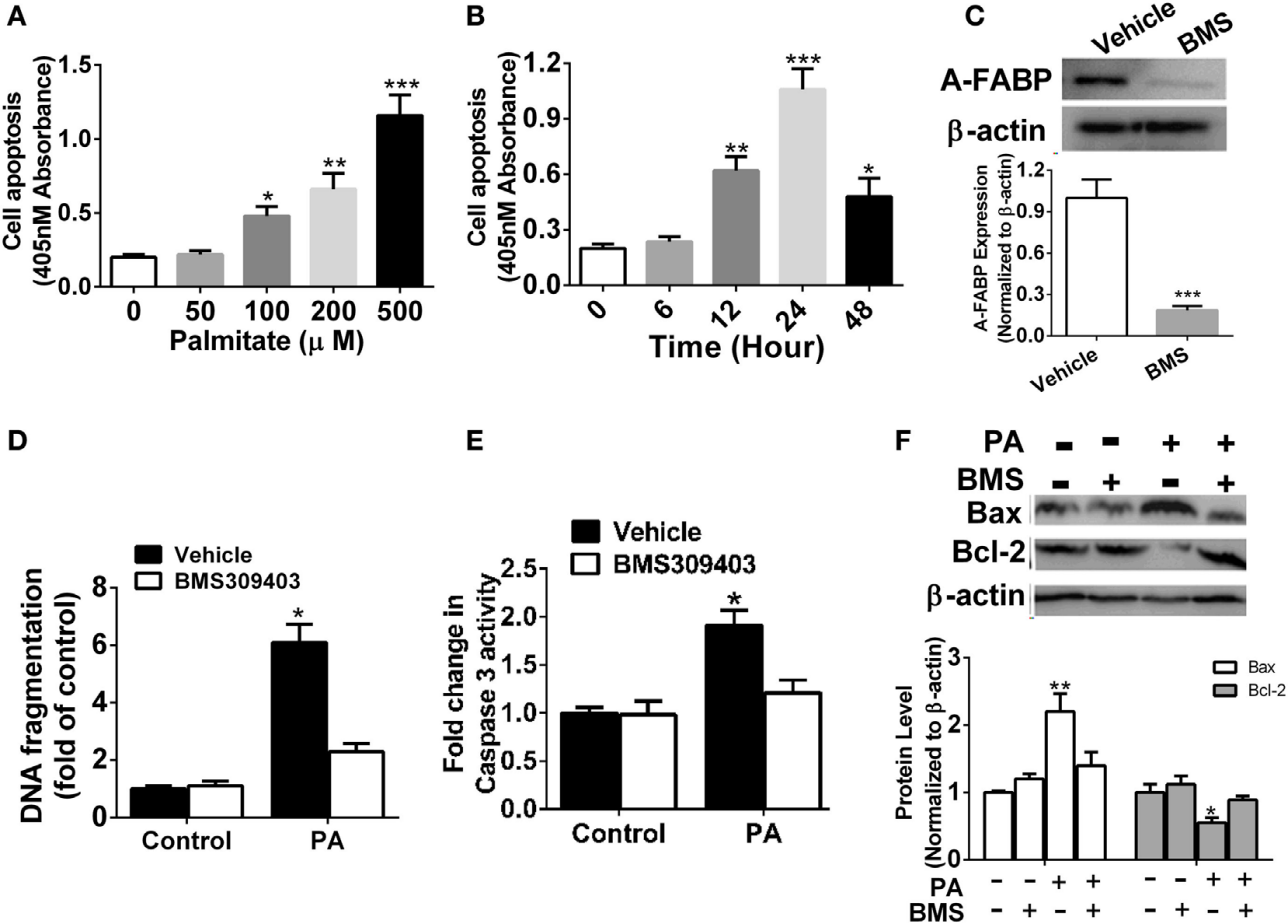
Adipocyte fatty acid-binding protein (A-FABP) inhibition with BMS309403 reduces macrophage apoptosis. **(A)** Quantitative analysis of cell apoptosis as determined by DNA fragmentation at 24 h after treatment with different concentrations of palmitate (PA). **(B)** Time course of cell apoptosis after 500 µM PA treatment. **(C)** Effect of BMS309403 on A-FABP expression induced by PA as determined by Western blot (upper panel) and its semiquantitative analysis (lower panel). **(D)** Effects of BMS309403 on cell apoptosis in Thp-1 cells after 500 µM PA treatment for 24 h. **(E)** Quantitative analysis of effects of BMS309403 on caspase-3 activity in Thp-1 cells treated with 500 µM PA for 24 h. **(F)** Effects of BMS309403 treatment on Bax and Bcl-2 expression in Thp-1 cells treated with 500 µM PA for 24 h. *P < 0.05, **P < 0.01, ***P < 0.001 versus vehicle control (*n* = 3–5 for each group).

It was previously reported that PA may induce macrophage apoptosis (5). Our results demonstrated a robust time- and dose-dependent increase in apoptosis in the Thp-1 cells treated with PA, as evidenced by DNA fragmentation assayed by cell death enzyme-linked immunosorbent assay (ELISA) (Figures 2A,B). To further explore the possible role of A-FABP in macrophage apoptosis induced by PA, we used the specific A-FABP blocker, BMS309403, to inhibit the activity of A-FABP in the Thp-1 cells. BMS309403 is an aromatic biphenyl azol compound that competes with fatty acids for the binding pocket of A-FABP with high specificity. As shown in Figure 2C, BMS309403 treatment rendered a marked decrease (≈95%) in A-FABP protein expression induced by PA, compared with the vehicle treatment with the PA addition. After BMS309403 treatment, PA-induced macrophage apoptosis was greatly inhibited, as evidenced by cell death analysis (Figure 2D). To further confirm whether blocking A-FABP could inhibit the apoptotic effect of PA on macrophages, we examined caspase 3 activity, the downstream signaling of apoptosis (Figure 2E). BMS309403 treatment remarkably inhibited the caspase 3 activity induced by PA treatment. Bcl-2 and Bax are two critical proteins in the mitochondrial-related apoptosis pathways, with anti-apoptotic and pro-apoptotic functions, respectively. As shown in Figure 2F, blocking A-FABP upregulated Bcl-2 but downregulated Bax in macrophages treated with PA. These findings suggested that A-FABP contributed to macrophage death through the mitochondrial apoptosis pathways.

### Effect of A-FABP Inhibition on Mitochondria Numbers and the Loss of Transmembrane Potential following PA Treatment

**Figure 3:**
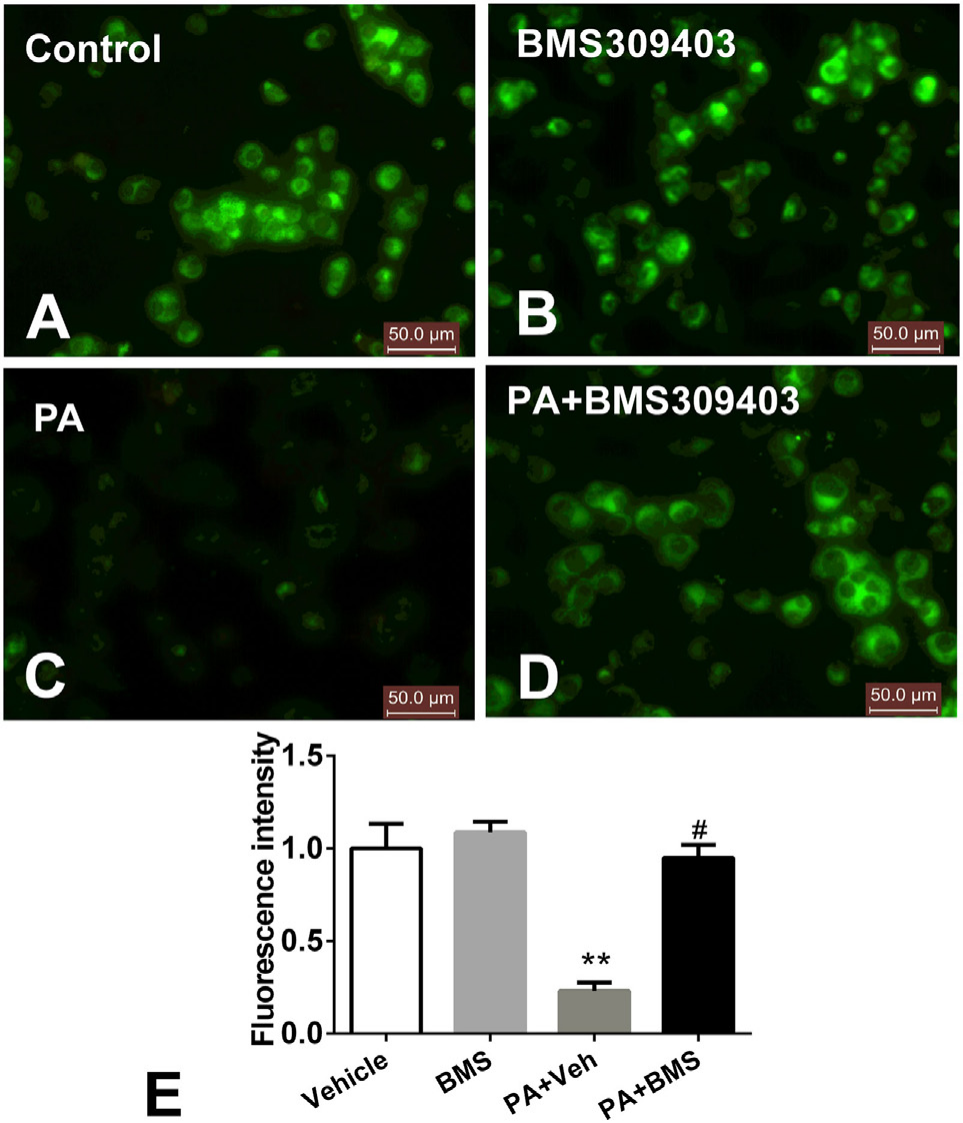
Adipocyte fatty acid-binding protein (A-FABP) inhibition with BMS309403 restores the palmitate (PA)-induced mitochondria number loss, determined by MitoTracker staining in macrophages. Thp-1 cells were pretreated with 50 µM BMS309403 for 2 h followed by stimulation with 500 µM PA. **(A–D)** Representative images of MitoTracker Green fluorescence in Thp-1 cells treated with **(A)** vehicle, **(B)** BMS309403, **(C)** 500 µM PA, **(D)** 500 µM PA combined with BMS309403. **(E)**. Semiquantitative analysis of MitoTracker staining intensity. Data represent the mean of three separate experiments. Scale bar = 50 µm. **P < 0.01 versus control, ^#^*P* < 0.05 Vehicle versus BMS309403.

**Figure 4:**
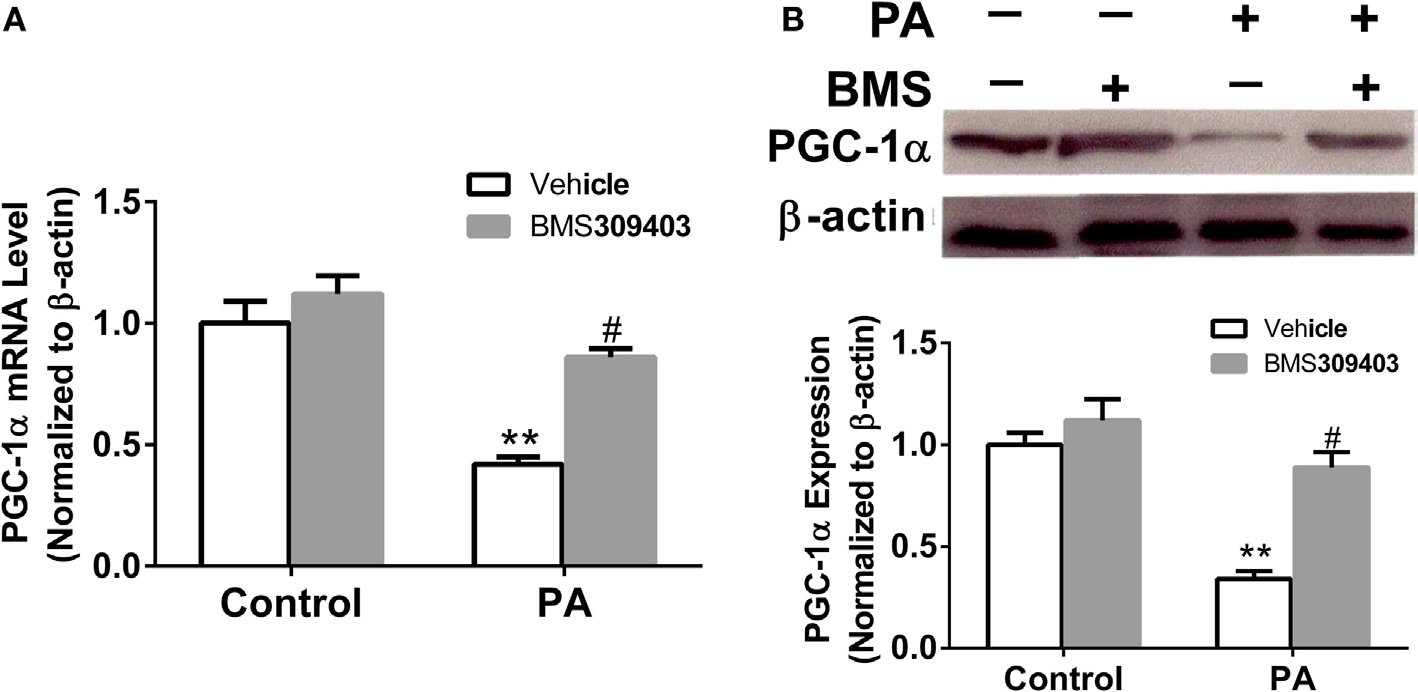
Adipocyte fatty acid-binding protein (A-FABP) inhibition with BMS309403 restores the expression of PGC-1α in macrophages. Thp-1 cells were pretreated with 50 µM BMS309403 for 2 h followed by 500 µM palmitate (PA) treatment. **(A)** Quantitative analysis of A-FABP mRNA level determined by real-time polymerase chain reaction. **(B)** Western blot of PGC-1α (top panel) and its semiquantitative analysis (lower panel). **P < 0.01 versus control, ^#^*P* < 0.05 Vehicle versus BMS309403 (*n* = 4 for each group).

The abovementioned results show that A-FABP may be involved in mitochondrial dysfunction and the apoptosis pathway induced by PA; therefore, we investigated the effect of A-FABP on mitochondria numbers following treatment with PA, *via* measurement with MitoTracker Green. As shown in Figure 3, PA dramatically decreased the number of mitochondria (Figures 3C,E). In contrast, blocking A-FABP with BMS309403 restored mitochondria numbers (Figures 3D,E) indicating that A-FABP activation by PA reduces the number of mitochondria. It is well known that PGC-1α regulates mitochondrial biogenesis (27) and one recent study showed that the overexpression of A-FABP reduced the expression of PGC-1α in adipocytes (21); therefore, we examined whether A-FABP inhibition could attenuate the effect of PA on PGC-1α in macrophages. As shown in Figure 4, PA reduced the expression of PGC-1α mRNA and protein. These findings suggest that PA inhibited mitochondrial biogenesis.

**Figure 5:**
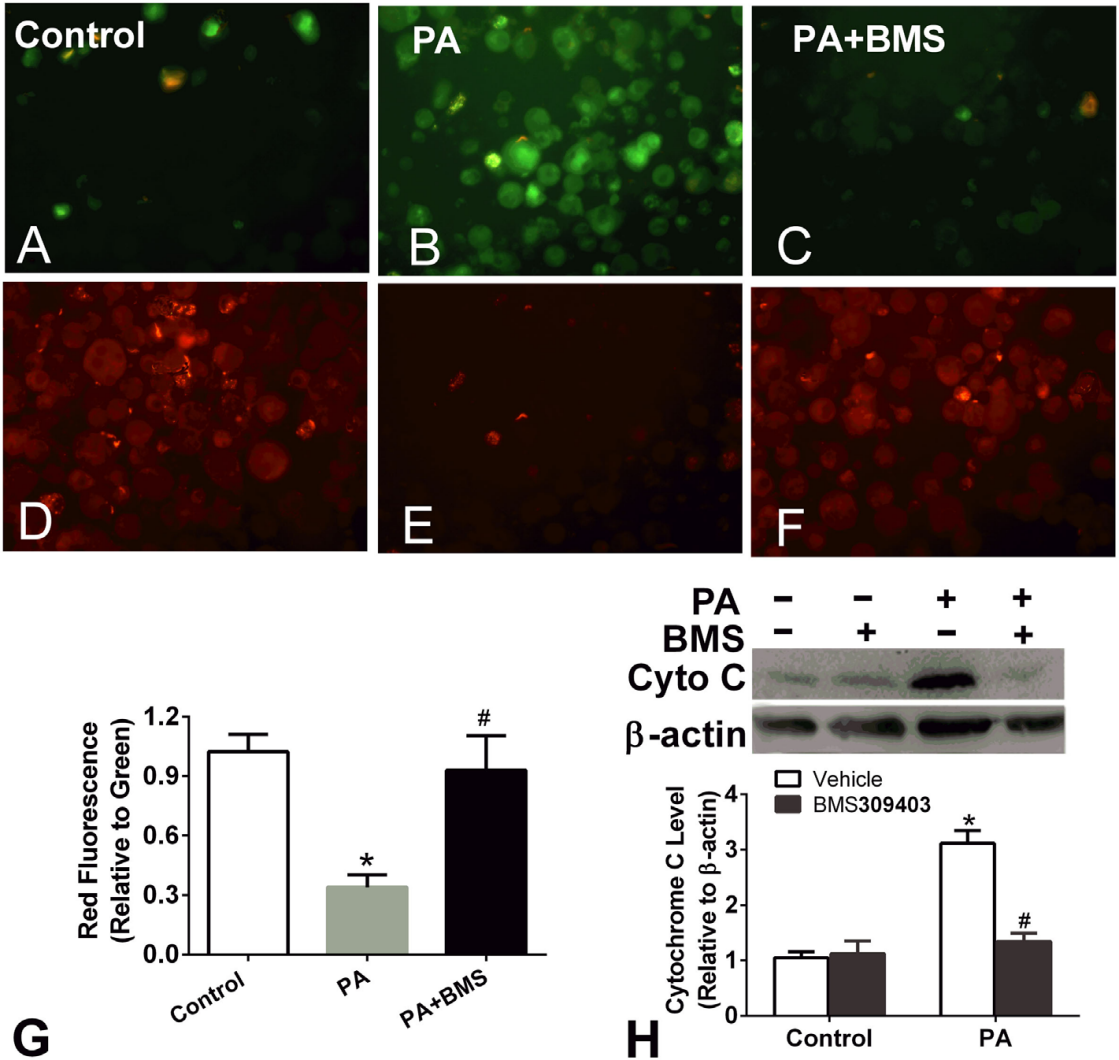
Adipocyte fatty acid-binding protein (A-FABP) inhibition rescues mitochondrial membrane potential upon palmitate (PA) treatment. **(A–C)** Images of the monomers (green fluorescence) of Thp-1 cells indicating the presence of depolarized mitochondria (apoptotic cells) after PA or/and BMS309403 treatment. **(D–F)** Image of the aggregates (red fluorescence) indicating the functional polarized mitochondria after PA and/or BMS309403 treatment. Scale bar represents 20 µm **(G)** Semiquantitative analysis of changes in Δψm in panels **(A–F)**. **(H)** Expression of cytochrome *c* in Thp-1 cells with 50 µM BMS309403 treatment for 2 h followed by 500 µM PA treatment. *P < 0.05 versus control, ^#^*P* < 0.05 Vehicle versus BMS309403 (*n* = 3–4 for each group).

Treatment of macrophages with fatty acids is associated with decreased mitochondrial transmembrane potential. The loss of Δψm and the release of cytochrome *c* into the cytosol are prominent features of the mitochondrial apoptosis pathway; therefore, we next performed a JC-1 assay to determine the Δψm of PA-treated macrophages and the effect of A-FABP inhibition on Δψm. PA treatment greatly reduced the Δψm, suggesting that PA-induced mitochondrial dysfunction (Figures 5A–G). BMS309403 treatment totally restored the Δψm, indicating that blocking A-FABP inhibited PA-induced mitochondrial dysfunction (Figures 5A–G). Similarly, PA treatment resulted in cytochrome *c* release into the cytosol, as indicated by Western blotting. The upregulation of cytochrome *c* in the cytosol was totally blocked by the A-FABP inhibitor (Figure 5H).

### Effect of Inhibition of A-FABP from PA Treatment on Mitochondrial Dysfunction

**Figure 6:**
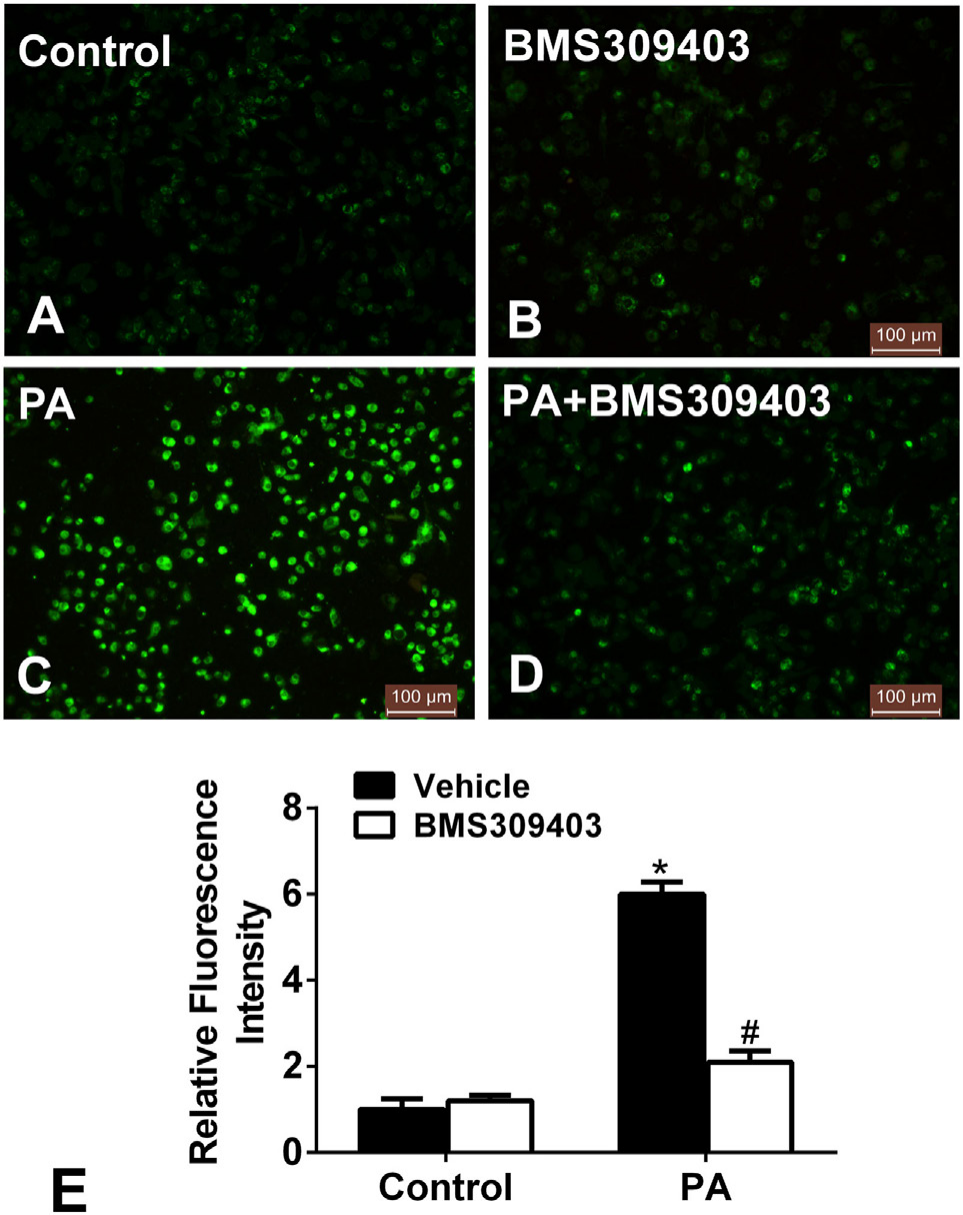
Adipocyte fatty acid-binding protein inhibition with BMS309403 reduces the increased production of reactive oxidative species (ROS) in macrophages induced by palmitate (PA) treatment. **(A–D)** Representative fluorescent images of intracellular ROS production treated by PA and/or BMS309403 in Thp-1 cells, detected by the fluorescent probe H2DCFDA. **(E)** Semiquantitative analysis of ROS generation in Thp-1 cells after treatment with PA and/or BMS309403. *P < 0.05 versus control, ^#^*P* < 0.05 Vehicle versus BMS309403 (*n* = 4 for each group).

We further examined whether blocking A-FABP could attenuate the mitochondrial dysfunction from PA treatment. Given that mitochondrial dysfunction would result in the production of ROS, which reciprocally exacerbated mitochondrial injury and damage, the production of ROS was measured. As shown in Figure 6, PA treatment increased the fluorescence intensity, strongly suggesting the production of ROS (Figures 6C,E). However, BMS309403 totally inhibited the increased fluorescence upon PA treatment, indicating that blocking A-FABP inhibited PA-induced ROS production (Figures 6D,E).

**Figure 7:**
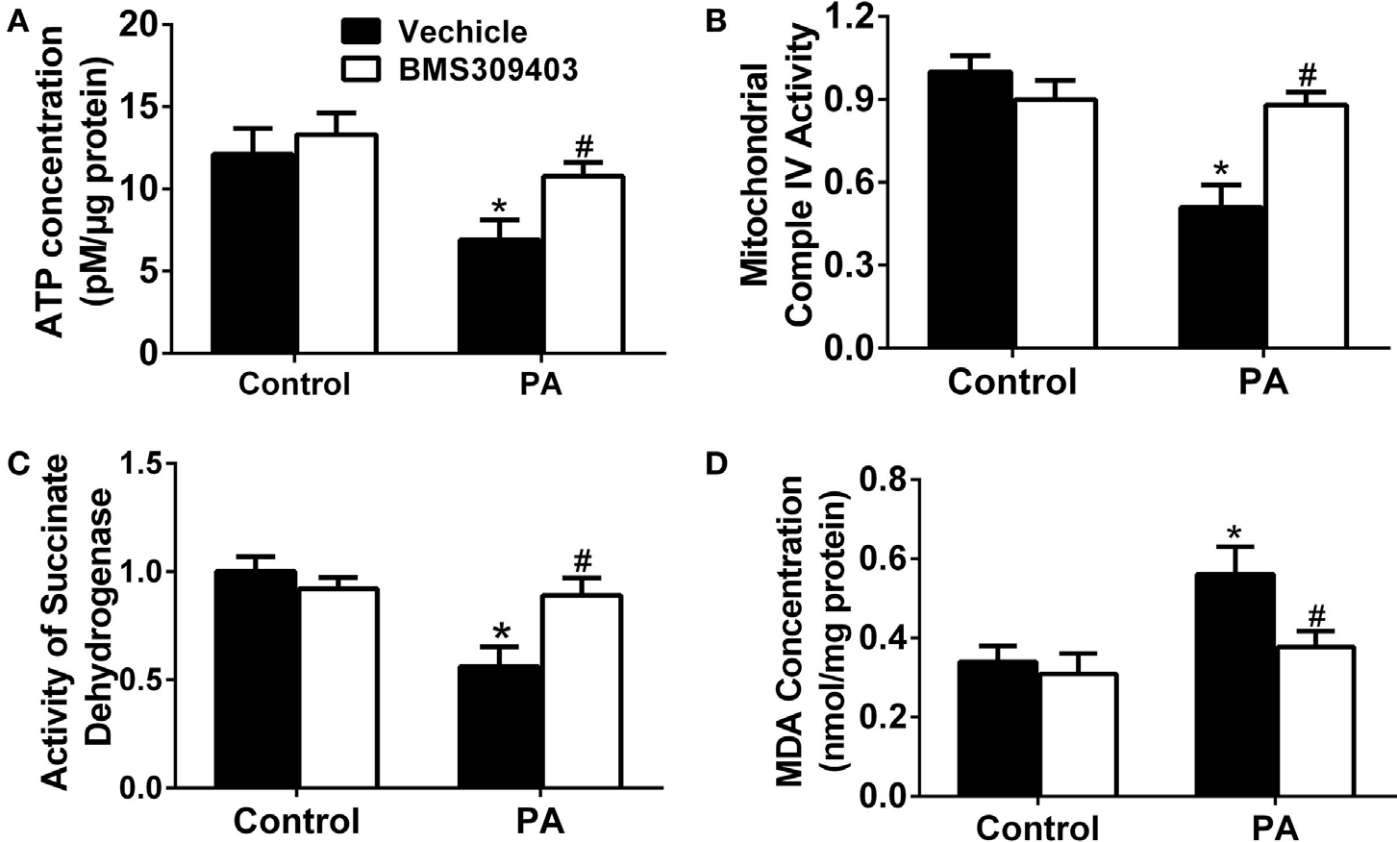
Adipocyte fatty acid-binding protein inhibition with BMS309403 attenuates palmitate (PA)-induced mitochondrial dysfunction in macrophages. Thp-1 cells were pretreated with 50 µM BMS309403 for 2 h followed by 500 µM PA treatment. At 24 h after treatment, adenosine triphosphate (ATP) level **(A)**, mitochondrial complex IV activity **(B)**, succinate dehydrogenase activity **(C)**, and malondialdehyde (MDA) concentration **(D)** were measured and quantified. *P < 0.05 versus control, ^#^*P* < 0.05 Vehicle versus BMS309403 (*n* = 3–4 for each group).

To further address the role of A-FABP in PA-induced mitochondrial dysfunction, we measured the adenosine triphosphate (ATP) concentration upon PA treatment followed by BMS309403 treatment. As shown in Figure 7A, PA treatment greatly reduced the ATP concentration, whereas BMS309403 reversed the decreased ATP concentration. Similarly, mitochondrial complex IV activity (Figure 7B) and succinate dehydrogenase (SDH) activity (Figure 7C), both of which are inhibited after mitochondria injury by PA, were restored after inhibiting A-FABP with BMS309403. Finally, the concentration of malondialdehyde (MDA), a marker reflecting the injury by ROS, was activated by PA treatment. BMS309403 remarkably inhibited the increase in MDA concentration upon PA treatment (Figure 7D). Taken together, these findings strongly indicate that PA results in mitochondrial dysfunction and ROS production through the activation of A-FABP.

## Discussion

There are two interesting findings in this study. First, consistent with previous studies in other types of cells, PA treatment resulted in macrophage apoptosis, ROS generation, and mitochondrial dysfunction. More importantly, this study provides the evidence that the lipid chaperon A-FABP mediates PA-induced mitochondrial dysfunction and the apoptosis pathway in macrophages. Thus, this study links the proatherosclerotic effects of A-FABP, saturated fatty acids, and macrophage apoptosis, and it sheds some light on the mechanisms of the important role A-FABP has in the pathogenesis of atherosclerosis.

Long-term exposure to a high level of saturated FFAs is considered to be a deleterious factor in fostering the development of atherosclerosis and cardiovascular disease. In contrast, unsaturated FFAs, such as oleate and linoleate, are substantially less cytotoxic, even protecting from PA-induced apoptosis or insulin resistance in some cell lines (28, 29). Therefore, this study examined the effect of PA on macrophage apoptosis, with a range of concentrations from 50 to 500 µM. The results showed that PA treatment ranging from 100 to 500 µM resulted in Thp-1-derived macrophage apoptosis. Consistent with our study, the apoptotic effect of PA at 500 µM was also observed in bone marrow-derived mouse macrophage lines (19). This suggests that a high level of circulating PA may exacerbate plaque vulnerability and the progress of atherosclerosis through its action on macrophage apoptosis.

It is believed that mitochondrial dysfunction resulting from the lipotoxicity of FFAs is a critical mechanism in the promotion of atherosclerosis (30–32). Indeed, mounting evidence suggests that the mitochondria play a crucial role in innate immune systems, and in particular, regulating macrophage functions and polarization in tissue injury, pathogens, and inflammation. However, the association between mitochondrial dysfunction in macrophages and lipotoxicity of FFAs during atherosclerosis remains unknown. Several studies found PA-induced mitochondrial dysfunction, apoptosis, and the inhibition of insulin signaling in skeletal muscle cells (7, 9). PA also induced endothelial cell apoptosis by ROS production, which was alleviated by silencing the Kv1.5 protein or *via* treatment with adiponectin (33, 34), but extensive exploration in mitochondria dysfunction is lacking. In this study, PA treatment in the Thp-1 macrophage induced the reduction of mitochondria numbers and Δψm, remarkable ROS release, and impairment of ATP production, decreasing mitochondrial complex IV activity, and SDH activity. These findings suggest that PA treatment induces mitochondrial dysfunction in macrophages. Excessive ROS may induce lipid peroxidation, as indicated by the remarkable increase of MDA in our study. Mitochondrial dysfunction and excessive ROS may also subsequently activate the mitochondrial apoptosis pathways (35). For the latter, cytochrome *c* is released to the cytoplasm because of the high permeability of the mitochondrial outer membrane. Cytochrome *c* assembles procaspase-9 and activates caspase-3 in succession, finally inducing apoptosis (36). Collectively, our results showed that PA treatment-induced mitochondrial dysfunction and ROS generation, both of which subsequently induce macrophage apoptosis.

Another major finding in this study is that the upregulation of A-FABP plays a critical role in PA-induced mitochondrial dysfunction and oxidative stress. In this regard, in a 10-year prospective study following 1,069 patients with prevalent coronary heart disease, A-FABP significantly predicted major secondary cardiovascular disease events, such as cardiovascular death and nonfatal myocardial infarction (37). In addition, ablation of the A-FABP gene in apolipoprotein E-deficient mice dramatically protects against atherosclerosis in the disease progress, and A-FABP exerts strong proatherosclerotic effects mainly through its actions on the macrophage (17, 38). In this study, PA treatment with a concentration of 50 µM upregulated the expression of A-FABP and induced a fourfold increase in the A-FABP gene level at 500 µM. Given that 500 µM PA may be more relevant to the clinical concentration of FFAs in patients with atherosclerosis and diabetes, it is assumed that A-FABP may be increased in atherosclerosis and diabetes patients, and this warrants further study. In this study, we used BMS309403 to inhibit the function of A-FABP. BMS309403 is a biphenyl azole inhibitor against A-FABP and highly effective for the treatment of both atherosclerosis and type 2 diabetes mellitus in mouse models (39). After blocking A-FABP by BMS30403, the PA treatment-induced macrophage mitochondrial dysfunction and apoptosis indexes were dramatically inhibited. These findings strongly suggested that A-FABP regulates PA-induced mitochondrial dysfunction and apoptosis in macrophages.

Adipocyte fatty acid-binding protein has a high affinity for binding and delivering FFAs to the mitochondria and other intracellular organelles and thus regulates lipid metabolism and inflammation (16). It is well known that increased fatty acid oxidation alleviates lipotoxicity by reducing intracellular levels of fatty acids. The overexpression of A-FABP may compromise fatty acid oxidation and reverse an enhancement of fatty acid oxidation induced by leptin in muscle cells and adipocytes, respectively (21, 40). Thus, the upregulation of A-FABP induced by PA seen in this study may impair fatty acid oxidation and render oxidative stress, both of which would result in mitochondrial dysfunction and apoptosis. Supporting this assumption, BMS309403 not only inhibited excessive ROS production as indicated by the decreased perioxidative marker MDA and generation of ROS but also attenuated PA-induced mitochondrial injury as indicated by reduced mitochondrial number, Δψm impairment, and restoration of decreased mitochondrial respiratory chain activity.

**Figure 8:**
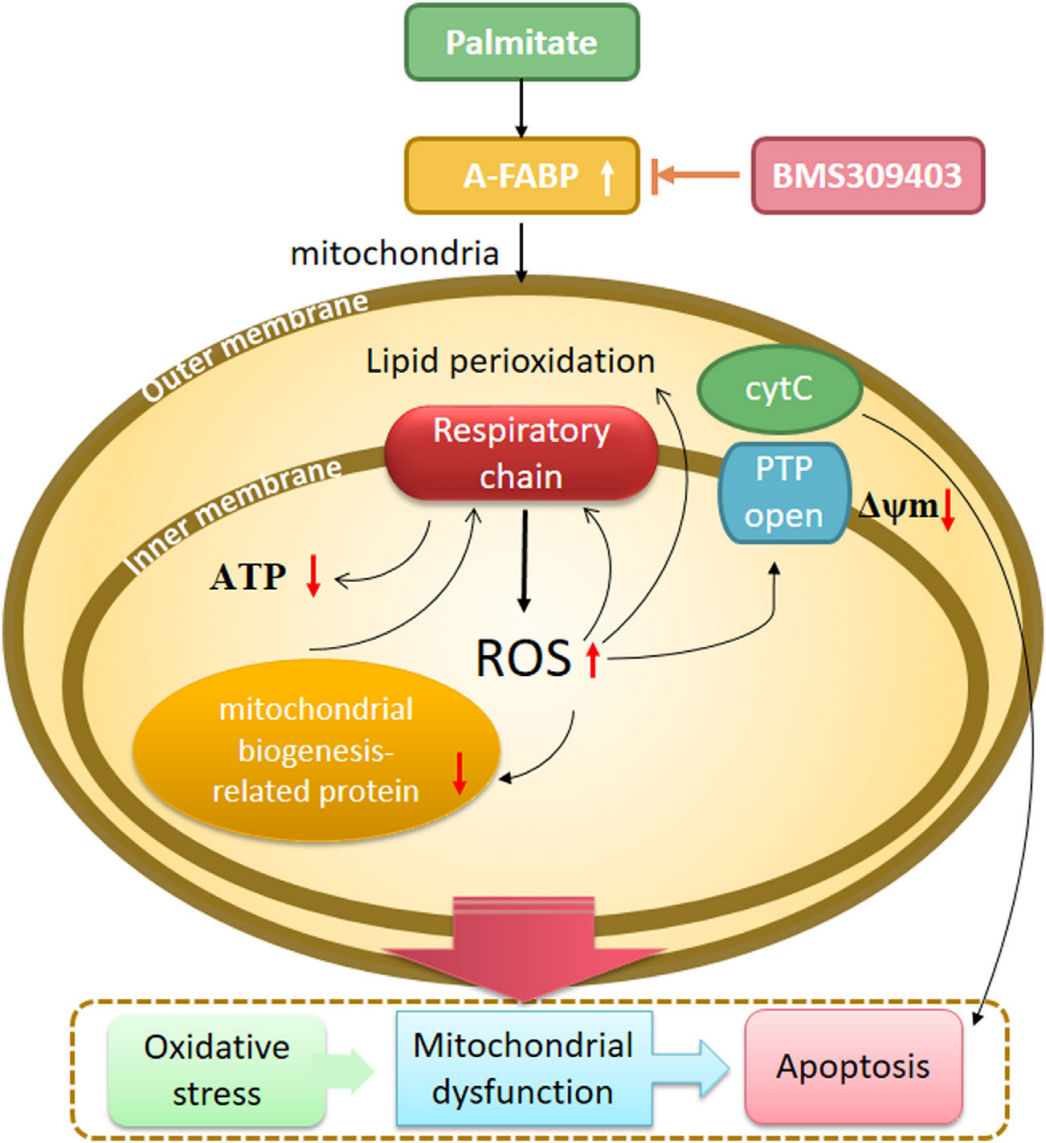
A proposed model of adipocyte fatty acid-binding protein (A-FABP)-mediated mitochondrial dysfunction and apoptosis induced by palmitate (PA) in macrophages. High exposure of PA leads to oxidative stress, indicated by excessive reactive oxidative species (ROS) generation originated from electron leakage of respiratory chain and subsequent lipid perioxidation. PA causes impaired mitochondrial biogenesis including reduced mitochondrial number and expression of mitochondrial biogenesis-related protein PPARγ coactivator 1-α. Mitochondrial function is also widespreadly compromised by PA as indicated by impaired adenosine triphosphate (ATP) generation, downregulated Δψm, and cytochrome *c* release. All of these eventually result in macrophage apoptosis through the activation of caspase 3. The inhibition of A-FABP with BMS309403 protects against mitochondrial dysfunction and apoptosis induced by PA.

Drawing together the findings of our study, we hypothesized that A-FABP serves as a linker of metabolites, apoptosis, and mitochondrial function through its effects on fatty acid oxidation in macrophages (Figure 8). In this scenario, PA overload increases A-FABP expression, and the increased A-FABP binds the saturated FFAs and delivers them to the mitochondria, along with impaired FFA oxidation. The FFAs would result in ROS production and impair mitochondrial dysfunction, leading to the release of cytochrome *c* into the cytoplasm, activation of caspase-3 and -9, and reduced ATP synthesis and mitochondrial biogenesis. Blocking A-FABP is potentially therapeutic for A-FABP-mediated lipotoxicity after metabolic overload. Further studies are needed to investigate how A-FABP regulates mitochondrial function and apoptosis. Notably, A-FABP can modulate the activity of several transcription factors, including Janus kinase (JAK)2 and PPARγ. The abnormalities in JAK2/STAT3 signaling are involved in apoptosis, the mechanisms of which are partly through oxidative stress and the mitochondrial pathway. JAK2/STAT3 activation can attenuate mitochondrial oxidative damage induced by myocardial ischemia/reperfusion injury and maintain mitochondrial function. Further investigations are warranted to explore the involvement of JAK2/STAT3 signaling in modulating A-FABP-mediated mitochondrial dysfunction and apoptosis induced by PA in macrophages.

The study has certain limitations. The *in vitro* environment may not mimic the *in vivo* system and clinical settings. However, previous studies have found increased FFA levels ranging from 440 to 960 µM in subjects with unstable coronary artery disease, and from 520 to 1,160 µM in men with ischemic heart disease (3, 4). Because PA accounts for ~30% of total plasma FFAs (41) and increases in stress states, following exercise or after a standardized dinner (42–45), PA levels could reach up to 500 µM in this type of patient. Moreover, another clinical study showed that the plasma PA concentration rose up to 526.5 ± 245.7 µM in type 2 diabetes mellitus patients (33). Thus, the 500 µM concentration of PA used in this study may be correlated with the clinical concentration. In addition, BMS309403 was used to inhibit A-FABP and investigate its effect on PA-induced macrophage apoptosis. It should be noted that BMS309403 may have some other pharmacological functions. In this regard, BMS30403 has been reported to stimulate glucose uptake *via* activation of the AMP-activated protein kinase signaling pathway, an A-FABP-independent mechanism in muscle cells (46). However, A-FABP is highly expressed in adipocytes and macrophages, with a much lower expression of A-FABP in the muscle cells. Thus, the effect of BMS309403 on PA-evoked macrophage mitochondrial dysfunction and apoptosis is mainly through the inhibition of A-FABP, if not entirely. Despite potential nonspecific effects, the easy administration of BMS309403 endows it as a drug to potentially prevent or treat atherosclerosis *via* targeting A-FABP.

In conclusion, our results established a link between saturated fatty acid overload, oxidative stress, mitochondrial dysfunction, and macrophage apoptosis. The discovery of the lipid chaperone A-FABP as an essential modulator regulating PA-induced oxidative damage, mitochondrial dysfunction, and subsequent macrophage apoptosis helps to extend our understanding of its contribution to the pathogenesis of atherosclerosis.

## Materials and Methods

### Reagents

The reagents Dulbecco’s modified Eagle’s medium (Invitrogen, Carlsbad, CA, USA), fetal bovine serum (FBS; Hyclone, Logan, UT, USA), PA, and penicillin–streptomycin (all from Sigma-Aldrich, St. Louis, CA, USA) were used. The A-FABP inhibitor BMS309403 was kindly provided by Professor Ai-Min XU from the Department of Pharmacology and Pharmacy, Li Ka Shing Faculty of Medicine, The University of Hong Kong. BMS309403 was synthesized as described previously (14) and dissolved in dimethyl sulfoxide.

### Cell Culture and Treatment

Cells from the Thp-1 human monocytic cell line (Shanghai Institute of Cell Biology, Chinese Academy of Sciences, Shanghai, China) were cultured in RPMI-1640 medium (Invitrogen) supplemented with 10% FBS and 1% P/S, at 37°C under 5% CO_2_. The cells were differentiated to macrophages by treatment with 100 nM phorbol-12-myristate-13-acetate (Sigma-Aldrich, St. Louis, MO, USA) in RPMI-1640 supplemented with 10% FBS and 1% P/S for 72 h. After 72 h of differentiation, the cells were treated with various concentrations of PA (0.05–0.5 mM) (Sigma-Aldrich) and dissolved in bovine serum albumin as the control for 24 h. The cells were used for the experiments.

### Analysis of Apoptosis

Apoptosis was assessed by several methods, including a cell death detection ELISA plus assay (Roche Molecular Biochemicals, USA) according to the manufacturer’s protocol. Briefly, cells were exposed to different concentrations of PA for different time periods and then incubated with a lysis buffer for 30 min at room temperature. After centrifugation, the supernatant (20 µL) was analyzed using ELISA. DNA fragmentation was determined by measuring the absorbance at 405 nm, with that at 490 nm used as a blank.

### Measurement of Reactive Oxidative Substances Levels

Production of intracellular ROS levels was measured with the fluorescent dye 2′,7′ dichlorodihydrofluorescein diacetate (H_2_DCFDA; Molecular Probes, Eugene, OR, USA). After treatment with fatty acids and/or inhibitors, the cells were incubated with 10 µM H_2_DCFHDA for 1 h at 37°C in the dark and analyzed with fluorescence microscopy.

### Measurement of Mitochondrial Membrane Potential

Disruption of the Δψm was measured using a MitoLight Mitochondrial Apoptosis Detection kit (Chemicon, Temecula, CA, USA) according to the manufacturer’s instructions. MitoLight solution was added to the cultured cells and incubated at 37°C in a 5% CO_2_ incubator for 15 min. Cells were then washed in PBS to remove excess MitoLight and examined under a fluorescence microscope (BX60, Olympus, Tokyo, Japan). Images were captured using a DP50 digital camera (Olympus). MitoLight aggregates in the mitochondria of healthy cells and fluoresces red. MitoLight cannot accumulate in the mitochondria of apoptotic cells; it remains as monomers in the cytoplasm and fluoresces green.

### Mitochondria Staining

Mitochondria of cells were stained using 0.5-µm MitoTracker Green (Molecular Probes, Invitrogen; 37°C, 5 min) and examined under a fluorescence microscope (BX60, Olympus).

### Caspase Activity Assay

A colorimetric assay was used according to the manufacturer’s instructions (Biovision, Mountain View, CA, USA) to measure the activity of caspase-3 in Thp-1 cells. Briefly, cells were lysed on ice for 10 min and centrifuged (10,000 *g*, 1 min). Cytosolic extracts (supernatant) were incubated at 37°C for 1–2 h with DEVD-pNA substrate or LEHD-pNA substrates (200 µM final concentration). Samples were analyzed by spectrophotometer (400 nm).

### Measurement of ATP Level

The intracellular ATP concentration was measured using an ATP colorimetric/fluorometric assay kit (Biovision). Cells (1 × 10^6^) were lysed in 100 µL of ATP assay buffer, homogenized, and centrifuged (15,000 *g*, 2 min, 4°C) to pellet insoluble materials. The supernatants and ATP assay buffer were added to 96-well plates with a final volume of 50 µL per well. The mixture was incubated at room temperature for 30 min and protected from light. The absorbance in the wells was measured at 570 nm using a microplate reader. The absorbance of the non-ATP control was subtracted from each reading.

### Mitochondrial Respiratory Activity

Macrophage mitochondria were harvested using the Cell Mitochondria Isolation kit (Beyotime, China). Cells were harvested and washed with cool-PBS twice, and then suspended in isolation buffer on ice for 15 min. The cells were homogenized, and then the homogenate was centrifuged at 1,000 *g* at 4°C for 10 min. The supernatant was then collected and centrifuged at 11,000 *g* for at 4°C 10 min. The mitochondria were harvested in the sediments for later experiments. The mitochondrial complex activities were determined using the Mito Complex IV and SDH Activity Assay kits (GenMed Scientifics Inc., China).

### MDA Measurements in Cellular Oxidant Injury

The level of lactate dehydrogenase and MDA released from Thp-1 cells into the culture media was analyzed with lactate dehydrogenase and MDA assay kits (Promega, Madison, WI, USA).

### Real-time Polymerase Chain Reaction (PCR)

Real-time PCR was performed to detect the gene expression of A-FABP, PGC-1α, and β-actin. The PCR sequence pairs were: AACCTTAGATGGGGGTGTCC and ATGCGAACTTCAGTCCAGGT (A-FABP); ACAGCCGTCGGCCCAGGTAT and GCCTCTCCCTTTGCTTGGCCC (PGC-1α); and GCCGACAGGATGCAGAAGGAG and AAGCATTTGCGGTGGACGATG (β-actin). PCR reaction was carried out using 15 ng of cDNA, 200 nM of each primer, and SYBR green PCR master mix (Roche, Germany). Cycling conditions included 10 min at 95°C followed by 40 cycles of 15 s at 95°C and 60 s at 60°C.

### Western Blotting Analysis

After treatment, protein extracts for total cellular fractions were isolated in cell lysis buffer (Cell Signaling Technology, Beverly, MA, USA) supplemented with 0.1 mg PMSF and a 1/100 dilution of protease and phosphatase inhibitor cocktails (Sigma). Scraped samples were then centrifuged at 10,000 *g* for 15 min at 4°C. The supernatants were used for Western blotting. Protein concentrations were measured using the protein dye microassay (Bio-Rad, Hercules, CA, USA). The proteins were separated on a 12% SDS-PAGE and then transferred onto PVDF membranes. The membrane was blocked with a solution of TBS and 5% fat-free milk for 1 h, then incubated overnight with anti-rabbit A-FABP (1:1,000), anti-rabbit Bax (1:2,000), anti-rabbit Bcl-2 (1:2,000), anti-rabbit PGC-1α (all from Cell Signaling Technology), and monoclonal anti-mouse β-actin (Santa Cruz Biotechnology, Heidelberg, Germany). The blot was then incubated with horseradish peroxidase-conjugated goat anti-rabbit IgG or goat anti-mouse IgG in TBS for 2 h at room temperature. The membrane was then exposed to film before development.

### Statistical Analysis

Data are expressed as mean ± SEM. Statistical analyses were performed using paired Student’s *t*-test or one-way analysis of variance followed by Bonferroni analysis where appropriate. Statistical significance was determined at the 0.05 level.

## Author Contributions

HL is involved in conduct of the study, data collection, data analysis, and manuscript preparation and revise, final approval of the version to be published, agreement to be accountable for all aspects of the work. YX is involved in conduct of the study, data collection, data analysis, and final approval of the version to be published, agreement to be accountable for all aspects of the work. LT is involved in conduct of the study, data collection, data analysis, and final approval of the version to be published, agreement to be accountable for all aspects of the work. FZ helps to do the mitochondrial-related experiments. GH helps with data analysis. J-MX helps to design and analyze the data, final approval of the version to be published, and agreement to be accountable for all aspects of the work. A-MX helps to generate and provide the A-FABP inhibitor BMS309403, data analysis, and final approval of the version to be published. R-PD is responsible for designing and interpretation of the work, data collection, data analysis, manuscript drafting and revising, final approval of the version to be published, agreement.

## Conflict of Interest Statement

The authors declare that the research was conducted in the absence of any commercial or financial relationships that could be construed as a potential conflict of interest.
